# Correlates of Obstetric Risk Perception and Recognition of Danger Signs in Kano, Northern Nigeria

**DOI:** 10.5334/aogh.376

**Published:** 2019-10-03

**Authors:** Zubairu Iliyasu, Hadiza S. Galadanci, Abubakar Abdurrahim, Abubakar Jibo, Hamisu M. Salihu, Muktar H. Aliyu

**Affiliations:** 1 Bayero University, NG; 2 Baylor College of Medicine, US; 3 Vanderbilt University Medical Center, Nashville TN, US

## Abstract

**Background:**

Risk perception and recognition of danger signs are important cues for accessing obstetric care. These measures are not well documented in many resource-limited settings, including northern Nigeria, a region with poor maternal health indices.

**Objective:**

To assess community level obstetric risk perception, danger sign recognition and their predictors in Kano, northern Nigeria.

**Method:**

This is a community-based cross-sectional study. Participants were surveyed using structured, pretested questionnaires. Knowledge of obstetric risk factors and danger sign recognition were analyzed, and their predictors modeled using logistic regression to generate adjusted odds ratios (AORs).

**Results:**

The obstetric risk factors identified by the 400 respondents included: maternal age (64.3%), history of abortion (37.0%), postpartum haemorrhage (36.0%), previous operative delivery (31.8%), and high parity (31.3%). The most frequently recognised danger signs during pregnancy were: vaginal bleeding (76.8%), seizures (44.5%), and severe abdominal pain (34.8%). Common intrapartum danger signs recognised included: severe bleeding (77.8%), seizures (55.5%), and loss of consciousness (38.3%). Severe bleeding (80.5%), seizures (42.0%), and high fever (28.5%) were the top three danger signs identified in the postpartum period. At multivariate level, respondent sex (female vs. male) (aOR = 3.10, 95% CI = 1.67–5.74), ethnicity (Yoruba vs. Hausa) (aOR = 7.53, 95% CI = 2.51–22.6), occupation (employed vs. unemployed) (aOR = 4.07, 95% CI = 1.87–8.84) and parity (≥5 versus 0) (aOR = 0.23, 95% CI = 0.06–0.92) predicted good obstetric risk perception. Participants’ ethnicity (Yoruba vs. Hausa) (aOR = 4.40, 95% CI = 1.10–19.2) and obstetric risk perception (good vs. poor) (aOR = 12.0, 95% CI = 6.8–21.2) predicted danger sign recognition.

**Conclusion:**

The perception of obstetric risk and recognition of danger signs were influenced by participant sex, parity, employment status, and ethnicity. Targeted communication strategies and community-based education are essential to enhance effective utilisation of emergency obstetric services.

## Background

Risk perception is a person’s expectancy of an adverse event [1]. The concept of risk during pregnancy has expanded following advances in knowledge and technologies employed during antenatal care, and increased community awareness [2]. A high-risk pregnancy is one in which the health or life of the mother, infant or both are jeopardized due to a disorder coincidental with or unique to pregnancy [3]. Reported obstetric risk factors include: poor obstetric history, short stature (<1.52 m), extremes of maternal age, very young maternal age (<15 years), and advanced maternal age (≥35 years). The latter is associated with several adverse pregnancy outcomes, including preterm birth, low birth weight, stillbirth, chromosomal defects, labour complications, and higher rates of cesarean section [4,5,6,7,8]. Other risk factors are nulliparity or grandmultiparity, size-date discrepancy, unwanted pregnancy, extreme social deprivation, history of preterm labour, multiple gestation, abnormal lie, and previous gynecologic surgery. Other risk factors include history of previous fetal abnormality, perinatal or neonatal death, chronic medical disorders, and infertility.

Danger signs are pointers to serious complications during pregnancy, labour, and postpartum. The common danger signs during pregnancy are vaginal bleeding, swollen hands and face, and blurred vision. During labour and childbirth, they include severe vaginal bleeding, prolonged labour, seizures, and retained placenta. Further, during the postpartum period, key danger signs include severe vaginal bleeding, loss of consciousness, and fever [9,10].

Researchers have suggested that pregnancy risk perception and danger sign recognition are critical influencers of health seeking behaviour, place of delivery and acceptance of medical interventions [11,12,13]. Risk perception is also central to behavioural health models, such as the health belief model [14], protection motivation theory [15], and prospect theory [16].

There have been reports on obstetric risk perception and danger sign recognition in Nigeria [17,18,20,27]. Studies in other parts of Africa found that the proportion of women with knowledge of at least one obstetric danger sign ranged from 53% in Tanzania to 82.5% in Ethiopia [24,28]. Similarly, among men, it varied from 42% in southern Ethiopia to 92.2% in Kenya [29,30]. In Asia, the corresponding figures among women were higher in Bangladesh (99.3%) than in Pakistan (64%) [32]. Studies in developing countries identified age at marriage, education, parity, extended family, place of residence, employment status, wealth, and previous hospital delivery as predictors of obstetric risk perception [27,37]. Similarly, age, parity, education, knowledge and practice of antenatal care, previous institutional delivery, and experiencing maternal death in an acquaintance predicted awareness of obstetric danger signs [27,36].

However, little research has been documented on community members’ obstetric risk perception and danger sign recognition in northern Nigeria, a region with one of the poorest maternal health indices in the country [18]. The objective of this study was to assess obstetric risk perception, recognition of pregnancy-related danger signs, and their predictors among adult community members in Kano metropolis, northern Nigeria.

## Methods

### Setting/Study population

The study was conducted in Kano metropolis, the second largest city in Nigeria. Kano State has over 13 million inhabitants [19], predominantly Hausa-Fulani Muslims. Other Nigerian ethnic groups are, however, well represented [20]. The study was carried out on adults (≥18 years) of both sexes resident in Tarauni Local Government Area (LGA) for at least one year. Temporary residents and visitors were excluded.

### Design and sampling

The survey was community-based and cross-sectional. The sample target (424) was obtained using Fisher’s formula, assuming obstetric risk perception of 50% and desired precision of 5% [21]. This number was increased by 10% to account for non-response. A multistage sampling method was used. In the first stage, one LGA (Tarauni) was selected from the eight LGAs in Kano metropolis using simple random sampling. In the second stage, five wards were selected from the 10 wards in the sampled LGA using the same method. One settlement was then selected from each ward followed by proportionate allocation. Finally, systematic sampling was used to select respondents in each sampled settlement. This was achieved by prior settlement mapping and house numbering. To identify the first house, a random number was selected between one and the settlement’s sampling interval. Subsequent houses were identified by adding the respective sampling interval. In each sampled house, one household was selected using a one-time ballot. All eligible adults in the selected household were approached to participate in the survey.

### Instrument description/Data collection

Informed consent was obtained from prospective respondents prior to the commencement of interviews. The consent form was translated into the local language (Hausa). Literate respondents indicated acceptance by signing the consent form, while non-literate participants used a thumbprint. Approval for the study was obtained from the Research Ethics Committee at Aminu Kano Teaching Hospital, Nigeria.

A pre-tested structured interviewer-administered questionnaire adapted from a previous study was used [22]. The questionnaire had four subsections; the first inquired about socio-demographic data, the second assessed perception of obstetric risk, while the third inquired about symptoms/signs respondents considered as danger signs during pregnancy, labour, and postpartum. The tool was pre-tested on a 10% sample in another location (Kumbotso). Content re-validation was confirmed by specialist obstetricians, while reliability was assessed using Cronbach’s alpha with a coefficient of 0.88. The questionnaire was professionally translated into the local language (Hausa). Accuracy was checked through independent back-translation. Interviews were conducted by trained medical students who spoke the local Hausa language.

### Measurements

Risk perception was measured by the respondents’ ability to mention attributes that may result in poor obstetric outcome related to maternal age, height, parity, previous operative delivery, neonatal death, hospitalization for pregnancy induced hypertension (PIH), previous post-partum hemorrhage, and poor obstetric history. Responses were scored and used to categorise respondents as having good or poor obstetric risk perception. Respondents who were able to mention at least two risk factors in Hobel’s abridged pregnancy risk assessment categories (obstetric history, medical history, physical, pregnancy-dependent) were considered to have good obstetric risk perception while those who couldn’t were categorised as having poor obstetric risk perception [23,24]. In addition, respondents were asked to mention danger signs that could trigger health-seeking behaviour during pregnancy, labour, or postpartum. The expected responses were severe headache, blurred vision, seizures, swollen hands and face, high fever, loss of consciousness, difficulty in breathing, severe weakness, severe abdominal pain, reduced or accelerated fetal movements, and breakage of water without labour. Similarly, danger sign responses during labour/delivery include: severe vaginal bleeding, severe headaches, seizures, high fever, loss of consciousness, labour lasting >12 hours, and retained placenta. Further, anticipated responses for postpartum danger signs were: severe vaginal bleeding, severe headaches, blurred vision, seizures, swollen hands/face, high fever, malodorous vaginal discharge, loss of consciousness, difficulty in breathing, and severe weakness and dizziness. These were also scored to categorise respondents into those with good or poor knowledge of obstetric danger signs. Based on ease of recognition and association with adverse outcomes, respondents who mentioned at least two key danger signs each during pregnancy (severe vaginal bleeding, swollen hands/face, blurred vision), labour/childbirth (severe vaginal bleeding, prolonged labour (>12 hours), seizures, retained placenta), and postpartum (severe vaginal bleeding, foul-smelling vaginal discharge, high fever) were considered to have good knowledge of danger signs of pregnancy; otherwise they were rated as having poor knowledge [22].

### Data analysis

Data were analyzed using SPSS version 21 [25]. Quantitative variables were summarized using mean and standard deviation or median and range based on distribution. Categorical variables were presented as frequencies and percentages. Pearson’s Chi-square or Fisher’s Exact test based on expected frequencies were utilized in bivariate analyses. Crude odds ratios (OR) were obtained using Stat Calc [26]. Multivariate logistic regression with variables that had *p* < 0.10 at bivariate level or conceptually important measures irrespective of their significance was used to identify independent predictors of obstetric risk perception and danger sign recognition. Adjustments were made for the confounding effects of age, sex, ethnicity, and education. All statistical tests were two-tailed with a type 1 error rate set at 5%.

## Results

### Socio-demographic characteristics

Out of 424 men and women approached, 400 agreed to participate, yielding a response rate of 94.3%. There were 201 males and 199 females—a sex ratio of approximately 1:1. The participants’ mean age (±SD) was 27.8 (±7.7) years and majority were Hausa/Fulani (85.8%) and Muslims (92.8%). More than a quarter of respondents (26.2%) were civil servants. Approximately 93% of participants had at least secondary school education (Table 1). More than half of respondents (56.0%) were single. The median number of children of ever married respondents was 3 (range: 1 to 8).

**Table 1:** Socio-demographic characteristics of respondents, Kano, Nigeria, 2016.

Characteristics	Frequency No. (%) N = 400

*Sex*	
Male	201 (50.2)
Female	199 (49.8)
*Age group*	
<20	9 (2.3)
20–29	253 (63.3)
30–39	103 (25.8)
≥40	35 (8.8)
*Ethnicity*	
Hausa	303 (75.8)
Fulani	40 (10.0)
Yoruba	31 (7.7)
Igbo	11 (2.8)
Others	15 (3.7)
*Religion*	
Islam	371 (92.8)
Christianity	29 (7.3)
*Education*	
No formal	12 (3.0)
Primary	17 (4.3)
Secondary	129 (32.3)
Post-Secondary	242 (60.5)
*Marital status*	
Single	224 (56.0)
Ever Married	176 (44.0)
*Occupation*	
Unemployed	27 (6.8)
Homemaker	100 (25.0)
Trading	81 (20.2)
Civil servant	105 (26.2)
Others+	87 (21.8)
*No. of children*	
0	294 (73.5)
1–4	79 (19.8)
≥5	27 (6.8)

+ Farmer, Tailor/Seamstress, Driver/Commercial tricylist, Barber/Hair dresser.

### Obstetric risk factor perception

The commonly mentioned obstetric risk factors perceived to portend adverse outcomes for mother and foetus were: maternal age (64.3%), parity (31.3%), history of abortion (37.0%), previous operative delivery (31.8%), and post-partum haemorrhage (36.0%) (Table 2). Overall, 42.0% and 58.0% of respondents had good and poor perception of obstetric risk factors, respectively (Table 3). The most recognised danger signs during pregnancy were: vaginal bleeding (76.8%), seizures (44.5%), and severe abdominal pain (34.8%). The most frequently recognised danger signs during labour included: severe vaginal bleeding (77.8%), seizures (55.5%), and loss of consciousness (38.3%). Further, severe vaginal bleeding (80.5%), seizures (42.0%), and high fever (28.5%) were the top three postpartum danger signs (Table 2). The proportion of respondents with good and poor knowledge of obstetric danger signs were 51.2% and 48.8%, respectively (Table 4).

**Table 2:** Obstetric risk perception and danger sign recognition, Kano, Nigeria, 2016.

Factors perceived to adversely affect the outcome of pregnancy	Frequency No. (%) N = 400

Maternal age	257 (64.3)
Previous history of abortion	148 (37.0)
Previous post-partum hemorrhage	144 (36.0)
Previous operative delivery	127 (31.8)
Number of previous births	125 (31.3)
Maternal weight	89 (22.3)
Previous neonatal death	85 (21.3)
Maternal height	71 (17.8)
**Recognition of danger symptoms/signs during pregnancy**	

Vaginal bleeding	307 (76.8)
Seizures	178 (44.5)
Severe abdominal pain	139 (34.8)
Loss of consciousness	112 (28.0)
Swollen hands and face	98 (24.5)
High fever	97 (24.3)
Severe headache	92 (23.0)
Water breakage before labour	90 (22.5)
Difficult breathing	88 (22.0)
Accelerated/reduced fetal movement	72 (18.0)
Blurred vision	62 (15.5)
Others (severe weakness, pallor etc.)	7 (1.8)
**Recognition of danger symptoms/signs during labour**	

Severe bleeding	311 (77.8)
Seizures	222 (55.5)
Loss of consciousness	153 (38.3)
High fever	111 (27.8)
Labour lasting >12 hours	104 (26.0)
Placenta not delivered 30 minutes after the baby	104 (26.0)
Severe headache	79 (19.8)
Others (hand prolapse, cord prolapse)	2 (0.5)
**Recognition of danger symptoms/signs after delivery**	

Severe bleeding	322 (80.5)
Convulsion	168 (42.0)
High fever	114 (28.5)
Loss of consciousness	112 (28.0)
Malodorous vaginal discharge	89 (22.3)
Difficult breathing	81 (20.3)
Blurred vision	80 (20.0)
Severe headache	73 (18.3)
Severe weakness/dizziness/pallor	68 (17.0)
Swollen hands/face	64 (16.0)

**Table 3:** Logistic regression model for predictors of obstetric risk perception, Kano, Nigeria, 2016.

Characteristics	^β^Good obstetric risk perception No. (%) N = 168	Poor obstetric risk perception No. (%) N = 232	Crude Odds Ratio (95% CI)	Adjusted Odds Ratio (95% CI)**	*P*-value

*Sex*					
Male	73 (43.5)	128 (55.2)	Ref		
Female	95 (56.5)	104 (44.8)	1.6 (1.07–2.39)	3.10 (1.67–5.74)	0.018†
*Age group*					
<20	3 (1.8)	6 (2.6)	Ref		
20–29	123 (73.2)	130 (56.0)	1.89 (0.46–7.73)	4.60 (0.75–28.4)	0.13
30–39	27 (16.1)	76 (32.8)	0.71 (0.17–3.04)	2.53 (0.37–17.3)	0.27
≥40	15 (8.9)	20 (8.6)	1.5 (0.32–6.9)	2.68 (0.35–20.5)	0.51
*Ethnicity*					
Hausa	114 (67.9)	189 (81.5)	Ref		
Fulani	15 (8.9)	25 (10.8)	0.99 (0.50–1.97)	1.22 (0.56–2.67)	0.42
Yoruba	25 (14.9)	6 (2.6)	6.9 (2.75–17.35)	7.53 (2.51–22.6)	0.026†
Igbo	6 (3.6)	5 (2.2)	1.99 (0.59–6.67)	3.34 (0.56–20.12)	0.63
Others	8 (4.8)	7 (3.0)	1.89 (0.67–5.36)	1.87 (0.48–7.32)	0.25
*Religion*					
Islam	150 (89.3)	221 (95.3)	Ref		
Christianity	18 (10.7)	11 (4.7)	2.41 (1.11–5.25)	1.01 (0.28–3.65)	0.16
*Education*					
No formal	4 (2.4)	7 (3.0)	Ref		
Primary	1 (0.6)	16 (6.9)	0.11 (0.01–1.16)	0.13 (0.01–1.74)	0.72
Secondary	43 (25.6)	87 (37.5)	0.86 (0.24–3.12)	0.61 (0.13–2.83)	0.55
Post-secondary	120 (71.4)	122 (52.6)	1.72 (0.49–6.03)	1.22 (0.26–5.66)	0.24
*Occupation*					
Unemployed	6 (3.6)	21 (9.1)	Ref		
Homemaker	31 (18.5)	69 (29.7)	2.04 (1.10–3.79)	1.41 (0.66–3.00)	0.44
Civil servant	47 (28.0)	58 (25.0)	0.72 (0.26–2.01)	0.83 (0.26–2.66)	0.36
Trading	23 (13.7)	58 (25.0)	1.13 (0.59–2.15)	0.68 (0.31–1.49)	0.53
Others+	61 (36.3)	26 (11.2)	5.91 (3.04–11.5)	4.07 (1.87–8.84)	0.004†
*Number of children*					
0	131 (78.0)	163 (70.3)	Ref		
1–4	33 (19.6)	46 (19.8)	0.89 (0.54–1.48)	0.69 (0.32–1.52)	0.15
≥5	4 (2.4)	23 (9.9)	0.22 (0.07–0.64)	0.23 (0.06–0.92)	0.012†

+ Farmer, Tailor/Seamstress, Driver/Commercial tricylist, Barber/Hair dresser. ** Logistic model includes the following variables: sex, age group, ethnicity, religion, education, occupation and number of children. † Significant at *p* < 0.05; OR: Odds Ratio, CI: confidence interval; Ref: reference group. ^β^ ≥2 risk factors in Hobel’s abridged pregnancy risk assessment categories (obstetric history, medical history, physical, pregnancy-dependent) = good obstetric risk perception; ≤1 risk factors in Hobel’s abridged pregnancy risk assessment categories (obstetric history, medical history, physical, pregnancy-dependent) = poor obstetric risk perception [23,24].

**Table 4:** Logistic regression model for predictors of danger sign recognition, Kano, Nigeria, 2016.

Characteristics	Good knowledge of obstetric danger signs. No. (%) N = 205	Poor knowledge of obstetric danger signs. No. (%) N = 195	Crude Odds Ratio (95% CI)	Adjusted Odds Ratio (95% CI)**	*P*-value

*Sex*					
Male	104 (50.7)	97 (49.7)	1.04 (0.70–1.54)	0.63 (0.36–1.09)	0.46
Female	101 (49.3)	98 (50.3)	Ref		
*Age group*					
<20	3 (1.5)	6 (3.1)	Ref		
20–29	126 (61.5)	127 (65.1)	1.98 (0.49–8.11)	1.62 (0.25–10.66)	0.31
30–39	55 (26.8)	48 (24.6)	2.29 (0.54–9.66)	3.03 (0.44–20.7)	0.27
≥40	21 (10.2)	14 (7.2)	3.00 (0.64–14.0)	1.89 (0.24–14.7)	0.16
*Ethnicity*					
Hausa	141 (68.8)	162 (83.7)	Ref		
Fulani	21 (10.2)	19 (9.7)	1.27 (0.66–2.46)	1.33 (0.60–2.93)	0.41
Yoruba	28 (13.7)	3 (1.5)	10.7 (3.19–36.0)	4.40 (1.10–19.2)	0.029†
Igbo	5 (2.4)	6 (3.1)	0.96 (0.29–3.20)	0.33 (0.038–2.91)	0.37
Others	10 (4.9)	5 (2.6)	2.30 (0.77–6.88)	1.65 (0.40–6.87)	0.18
*Religion*					
Islam	185 (90.2)	186 (95.4)	Ref		
Christianity	20 (9.8)	9 (4.6)	2.23 (0.99–5.04)	1.41 (0.31–6.42)	0.26
*Education*					
No formal	6 (2.9)	5 (2.6)	Ref		
Primary	3 (1.5)	14 (7.2)	0.18 (0.03–0.99)	0.20 (0.028–1.43)	0.13
Secondary	61 (29.8)	69 (35.4)	0.74 (0.21–2.54)	0.59 (0.14–2.44)	0.18
Post-Secondary	135 (65.9)	107 (54.9)	1.05 (0.31–3.54)	0.64 (0.15–2.67)	0.25
*Occupation*					
Unemployed	13 (6.3)	14 (7.2)	Ref		
Homemaker	35 (17.1)	65 (33.3)	1.23 (0.68–2.20)	0.78 (0.36–1.70)	0.36
Civil servant	61 (29.8)	44 (22.6)	0.82 (0.34–1.96)	0.90 (0.33–2.46)	0.27
Trading	43 (21.0)	38 (19.5)	0.48 (0.26–0.87)	0.35 (0.16–1.74)	0.23
Others+	53 (25.9)	34 (17.4)	1.38 (0.75–2.54)	0.38 (0.17–1.85)	0.37
*Obstetric risk Perception*					
Poor	68 (33.2)	164 (84.1)	Ref		
Good	137 (66.8)	31 (15.9)	10.7 (6.6–17.3)	12.0 (6.8–21.2)	<0.001†

+ Farming, Tailor/Seamstress, Driver/Commercial tricylist, Barbing/Hair dresser. ** Logistic model includes the following variables: sex, age group, ethnicity, religion, education, occupation and obstetric risk perception. † Significant at *p* < 0.05; OR: Odds Ratio, CI: confidence interval; Ref: reference group.

### Predictors of obstetric risk perception and danger sign recognition

At bivariate level, obstetric risk perception was significantly associated with respondent sex, age, ethnicity, religion, education, occupation, and number of children (*p* < 0.05). At multivariate level, respondent’s sex, ethnic group, occupation, and number of children remained significant predictors of obstetric risk perception (Table 3). After adjusting for other variables, female respondents had a greater than three-fold likelihood of good obstetric risk perception compared to males (aOR = 3.10, 95% CI = 1.67–5.74). Similarly, respondents of Yoruba ethnicity had >7 times the odds of good obstetric risk perception compared to their Hausa counterparts (aOR = 7.53, 95% CI = 2.51–22.6). Respondents engaged in other occupations had greater than four-fold chance of having good obstetric risk perception relative to unemployed respondents (aOR = 4.07, 95% CI = 1.87–8.84). Further, respondents with five or more children were 77% less likely to have good obstetric risk perception compared to nullipara (aOR = 0.23, 95% CI = 0.06–0.92).

Bivariate analysis also found significant association between obstetric danger sign recognition (during pregnancy, delivery, and postpartum) and ethnicity, religion, education, occupation, and obstetric risk perception. After adjusting for confounding using multivariate logistic regression, only ethnic origin and obstetric risk perception remained significant predictors of obstetric danger sign recognition. Specifically, there was a more than four-fold increased likelihood of recognising obstetric danger signs among Yoruba respondents compared to those of Hausa ethnicity (aOR = 4.40, 95% CI = 1.10–19.2). Similarly, respondents with good obstetric risk perception were more than 12 times likely to detect obstetric danger signs compared to their peers with poor obstetric risk perception (aOR = 12.0, 95% CI = 6.8–21.2) (Table 4).

## Discussion

Maternal age, parity, history of abortion, previous operative delivery, and post-partum haemorrhage were identified as obstetric risk factors by participants. Specifically, severe abdominal pain, vaginal bleeding, and seizures were perceived to portend danger during pregnancy. In addition to these, loss of consciousness was recognised as ominous during labour. Further, severe vaginal bleeding, seizures, and high fever were acknowledged as the top three danger signs during the postpartum period. Respondent’s sex, ethnic group, occupation, and parity predicted obstetric risk perception, while ethnic origin and obstetric risk perception predicted ability to recognise obstetric danger signs.

Not surprisingly, vaginal bleeding and seizures were frequently mentioned by our respondents as danger signs during pregnancy and puerperium. These results are similar to findings from previous studies among men in Kano, where sickness (48.1%), pregnancy while breastfeeding (26.5%), short pregnancy intervals (25.4%), younger mothers (23.7%), previous operative delivery (19.8%), and twin or higher order multiple pregnancies (1.8%) were considered as high risk. Similarly, 51.9%, 37.8%, 33.2%, 21.6% and 15.4% of men identified vaginal bleeding, seizures, loss of consciousness, pallor, and cessation of fetal movement as danger signs [40]. Higher proportions of women in rural (62.4%) and urban (68.4%) communities of Lagos, Nigeria had good knowledge of obstetric danger signs. Vaginal bleeding was the most commonly identified danger sign [41]. However, three-quarters of women in another study in suburban Lagos disagreed with vaginal bleeding as an obstetric danger sign. The respondents justified their position by the expectation that every pregnant woman would bleed during labour, and they had no way of ascertaining when blood loss becomes excessive [27]. Similar to our findings, slightly more than half (53%) of women studied in Tanzania knew at least one obstetric danger sign. Specifically, 26%, 23%, and 40% of them knew at least one danger sign during pregnancy, delivery, and puerperium, respectively [28]. A higher proportion of participants (82.5%) in a study in Tigray, Ethiopia knew at least two danger signs of pregnancy compared to 51.2% observed among our respondents [24]. However, only 42% of men were aware of danger signs of pregnancy in southern Ethiopia compared to 51.7% in our study [29]. In contrast, Kenyan men displayed good knowledge of obstetric danger signs, as 92.2%, 91.6%, and 90.4% of them recognised severe abdominal pain, absence of fetal movement, and long labour, respectively, as obstetric danger signs [30]. It is noteworthy that none of our respondents mentioned high blood pressure as a danger sign. This could be due to its asymptomatic nature. These results are in contrast with the more visible, frightening, and dramatic seizures of eclampsia mentioned by over half of the respondents.

A study in Bangladesh found that nearly all (99.33%) respondents recognised ‘water break’, severe nausea and vomiting (87.67%), and vaginal bleeding (85%) as prominent danger signs of pregnancy [31]. In contrast, a study in Pakistan reported poor knowledge of serious pregnancy-related complications. Participants in that study identified absent/decreased fetal movement (5%), premature uterine contractions (3%), premature rupture of membranes (3%), seizures (13%), obstructed labour (23%), and bleeding per vaginum (39%) as danger signs during pregnancy [32]. Considering the high proportion of births that occur at home in our study area [18], prompt recognition of danger signs during pregnancy, labour, and puerperium is critical in order to inform care seeking decisions. Well-informed women are likely to notify their partners and close confidants if they notice any deviation from the norm, which in turn could trigger remedial action.

Appropriate risk perception by pregnant women and their families is important, as exaggerated risk perception could also cause anxiety and trigger false alarms thereby overwhelming health care providers and de-sensitizing the response systems. Similarly, incongruence between health care professionals’ risk assessment and those made by women and their partners and differences in risk perception by socio-economic status, where women of higher socioeconomic stratum were reported to be more worried about pregnancy risk compared with those in the lower category (although risk was more prevalent in the latter) requires targeted communication [13]. It is important to bear in mind that risk can change suddenly and predictive utility of obstetric risk factors among high risk women varies. For instance, in Zimbabwe, a study found that 42.3% of cases of cephalopelvic disproportion could be predicted, compared with only 35.0% of those with post-partum haemorrhage, hence the need for individualized care during pregnancy, labour, and the postpartum [33].

Recent advances in obstetric management and technology has not decreased women’s obstetric risk perception [34]. Rather, the range of modern prenatal investigations, surveillance, medico-legal milieu, high-tech infertility treatment, and use of the internet and social media have all combined to heighten women and their partners’ anxiety about pregnancy in developed countries [35]. Whether this is the case in low-resource settings needs to be investigated. Stress, anxiety, and depression resulting from increased perception of risk during pregnancy could also have far reaching implications for the health of mothers, babies, families and the health system [35]. Conversely, women who downplay their risk status as a result of ignorance, non-utilization of antenatal care, and appropriate investigations, as is the case in low-resourced settings [36], require targeted information, education, and communication interventions.

Respondent’s sex, ethnic group, occupation, and parity predicted obstetric risk perception. Other researchers identified place of residence, employment status, wealth, and previous hospital delivery as the predictors [27]. Our finding of an inverse relationship between parity and obstetric risk perception in our sample is surprising, as previous reports indicate otherwise [17,43,44]. In Ethiopia, for example, women with high parity (4–6 pregnancies) were three times as likely to be aware of obstetric danger signs compared to those of lower parity [24]. That seems more logical, as one might expect that the more children one has, the more experience and greater obstetric risk perception the person would have. Our finding could be related to the peculiar cultural setting of northern Nigeria, where women of high parity tend to be over-confident and feel invulnerable. These highly parous women avoid health facilities, prefer to deliver at home as a mark of pride, and consider hospital delivery as a last resort [42,43]. They are, therefore, more likely to downplay the risks associated with pregnancy and delivery. On the contrary, women of low parity and their partners are more likely to be anxious about pregnancy and its outcome and are more likely to seek knowledge about danger signs. Health care workers should be aware of this apparent paradox and closely monitor women with high parity. The low proportion of antenatal clients reportedly informed about danger signs during antenatal care in the study area from previous studies (58.4%) [18] could partly explain the apparent disparity, and underscore the need for effective communication during pre-conception and prenatal care.

In contrast to our findings, the Lagos study earlier cited found that knowledge of antenatal care and experiencing maternal death in an acquaintance predicted awareness of obstetric danger signs [27]. The experience could be a constant reminder of obstetric risk. Similarly, in the health belief model, health literacy informs attitude and behaviour [14]. In Rural Tanzania, acquiring at least secondary education increased by six-fold the likelihood of obstetric danger sign awareness compared with those with no education at all. Other determinants were age, parity, antenatal visits, and previous institutional delivery [36].

A study in Nepal found that while women perceived some susceptibility to adverse events during pregnancy, they did not feel it was serious enough to necessitate biomedical care. Similar to our study setting, antenatal care attendance was higher than health facility births and postnatal attendance [37]. Further, education, parity, age at marriage, and extended family predicted risk perception [37]. Another study in Canada identified five factors as significant predictors of perception of pregnancy risk. These include: pregnancy-related anxiety, maternal age, medical risk, perceived internal control, and gestational age, accounting for 47% to 49% of the variance in risk perception [38].

While there are no certainties about the perinatal outcomes of pregnancies based on self or professional risk assessment, as apparently low risk pregnancies can result in poor outcomes and vice versa, some women take extreme measures to have what they perceive as normal deliveries. For instance, many Somalian women voluntarily decreased food intake during pregnancy in order to have a smaller foetus, an easier delivery and to avoid caesarean section [39].

In considering these findings, however, a few limitations merit mention. First, regarding the study setting and study participants, respondents were urban residents and were more likely to be better educated and hence more knowledgeable than their rural counterparts. Their obstetric risk perception is expected to be better than rural dwellers. Second, although individual interviews were conducted privately by trained interviewers from the same culture, social desirability bias cannot be ruled out, as respondents knew that the interviewers were medical students.

In conclusion, we found that perception of obstetric risk and recognition of danger signs were influenced by participant sex, employment status, and ethnicity among a cross section of respondents in Kano, northern Nigeria. The low obstetric risk perception and danger sign recognition require targeted information, community-based education and communication strategies to enhance timely utilization of emergency obstetric services.
